# Is Fluoride Blameless?—The Influence of Fluorine Compounds on the Invasiveness of the Human Glioma-like Cell Line U-87

**DOI:** 10.3390/ijms252312773

**Published:** 2024-11-27

**Authors:** Wojciech Żwierełło, Agnieszka Maruszewska, Marta Skórka-Majewicz, Agata Wszołek, Izabela Gutowska

**Affiliations:** 1 Department of Medical Chemistry, Pomeranian Medical University in Szczecin, 70-111 Szczecin, Poland; marta.skorka.majewicz@pum.edu.pl; 2 Department of Physiology and Biochemistry, Institute of Biology, University of Szczecin, 70-453 Szczecin, Poland; agnieszka.maruszewska@usz.edu.pl (A.M.); agata.wszolek@usz.edu.pl (A.W.)

**Keywords:** glioblastoma, fluoride, invasive potential, metalloproteinases

## Abstract

Glioblastoma remains one of the most treatment-resistant and malignant human cancers. Given the documented harmful effects of fluoride on the developing central nervous system and the rising incidence of brain tumors, especially among children, it is pertinent to explore the role of environmental toxins, including fluoride compounds, in the context of brain cancer. This study represents the first investigation into the influence of fluoride on mechanisms related to the invasiveness of human glioblastoma cells. We examined the effects of sodium fluoride (NaF) exposure on the migratory and invasive abilities of the U-87 human glioblastoma cell line, assessing levels of metalloproteinases MMP-2 and MMP-9 secreted by these cells. Additionally, the activation of metabolic pathways associated with invasiveness, including AKT and NF-κB, was analyzed. Our results suggest that the effects induced by NaF at physiologically high concentrations (0.1–10 µM) in U-87 glioblastoma cells may promote a pro-invasive phenotype.

## 1. Introduction

The debate surrounding the harmful effects of fluoride compounds has persisted for decades, with polarized views on fluoride’s role as an essential element and the extent of its toxicity to humans, particularly in the context of water fluoridation [1]. While fluoride deficiency can impair tooth enamel remineralization, support cariogenic oral microflora, promote tooth decay, and contribute to bone demineralization [2,3,4], the health benefits of fluoride appear to end there. Increasing evidence suggests that even minimal fluoride exposure may pose risks to human health, particularly in industrialized regions where environmental fluoride levels are elevated due to anthropogenic sources [2,5]. Fluoride distribution in soil, air, and water leads to accumulation in plants and animals, with artificial water fluoridation further affecting exposure levels [6,7,8,9].

Recent findings that fluoride can penetrate the blood–brain barrier (BBB) and alter nervous tissue structure and function are particularly concerning [10,11,12,13]. Fluoride exposure impacts neurotransmitter metabolism, receptor expression [14,15,16], brain energy metabolism [17], and amino acid and lipid metabolism [14,18], pointing to a potentially destructive role in the central nervous system (CNS). However, no studies have yet examined how fluoride might affect tumor development within the CNS, including gliomas.

Gliomas are primary brain tumors originating from glial cells [19]. Despite recent advancements in treating many tumor types, the survival rate for glioblastoma multiforme (GBM) patients remains at approximately 1.5 years [20]. Standard GBM treatment involves surgical resection followed by chemotherapy and radiotherapy, but the high invasiveness and infiltration of GBM cells present significant challenges [21]. GBM cells also traverse the extracellular matrix (ECM) with the aid of matrix metalloproteinases (MMPs), particularly MMP-2 and MMP-9, which are frequently overexpressed [22,23,24,25].

Fluoride compounds impact metabolic pathways, primarily those related to inflammation, such as NF-κB, Wnt, and AKT. Neuronal plasticity, associated with disruptions in the expression and activity of key metalloproteinases, may also be affected by fluoride exposure. These same pathways could theoretically contribute to the development of invasive potential in various types of tumors, including brain tumors [26]. Our hypothesis is that fluorine compounds crossing the blood–brain barrier can accumulate within gliomas and cause adverse effects for the patient. Potential alterations in the activity of key signaling pathways could contribute to the development of invasive potential. Therefore, we chose to use a human glioma-like cell line, U-87, expose it to physiological concentrations of fluoride, and analyze its invasive potential. These studies are the first to investigate the impact of fluoride on mechanisms related to the invasiveness of human glioblastoma cells.

## 2. Results

### 2.1. Cytotoxicity

**Figure 1:**
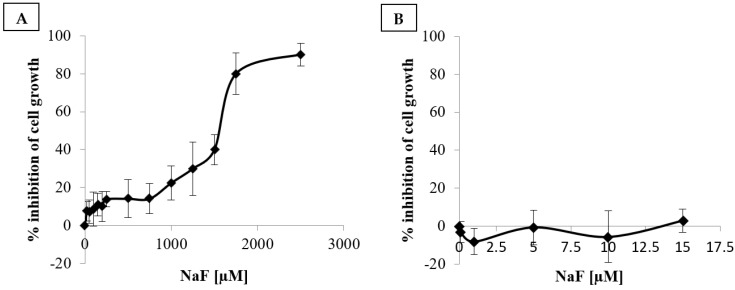
Cytotoxicity of NaF was assessed (**A**) at concentrations ranging from 0.1 to 2500 µM and (**B**) at concentrations ranging from 0.1 to 15 µM against U-87 cells. Results depict the percentage of growth inhibition relative to the control. Data represent the arithmetic mean with standard deviations, based on three biological and three technical replicates.

Cytotoxicity measurement using the MTT assay showed that the concentration of NaF required to achieve 50% inhibition of U-87 cells growth is IC_50_ = 1641 ± 71 µM (Figure 1A). Additionally, cytotoxicity measurements across the experimental concentration range indicated that NaF at 0.1–10 µM does not result in statistically significant inhibition of U-87 cell growth (Figure 1B).

### 2.2. Migration Potential of U-87 Cells

**Figure 2:**
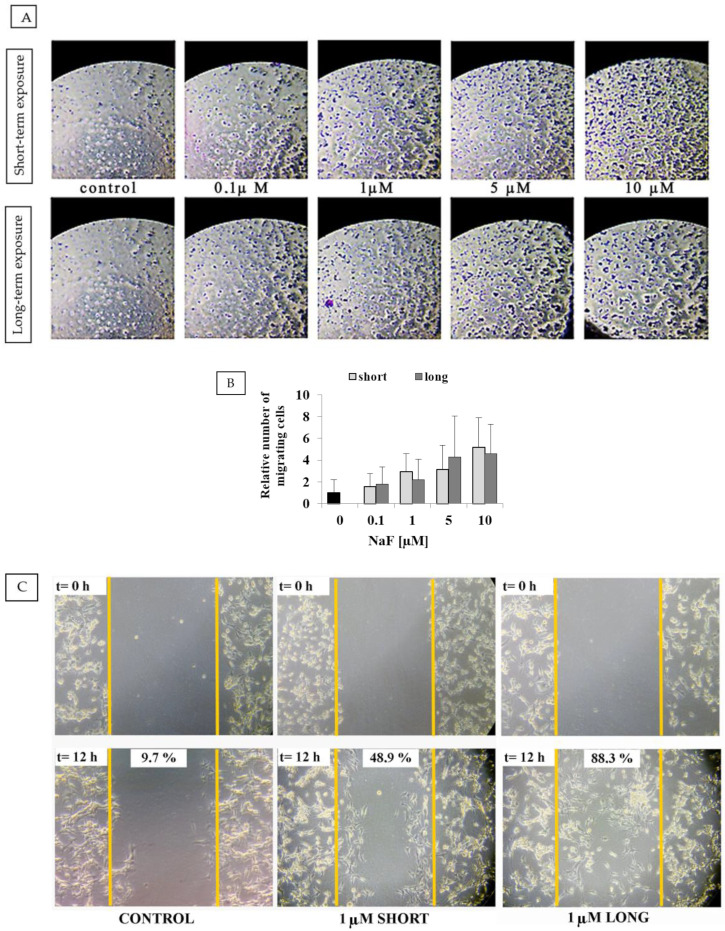
Effect of NaF on the migration of U-87 cells: (**A**) Representative microscopic image of cells on the basal side of the Boyden chamber after 12 h of incubation with NaF (0.1–10 µM), (**B**) relative number of migrating cells compared to control (control = 1), quantified in the field of view under the microscope in Boyden chambers (data represent the arithmetic mean with standard deviations), and (**C**) representative microscopic image of U-87 cells in the wound healing assay following 12 h of incubation with NaF (1 µM). Three biological replicates and three technical replicates were performed.

Analysis of migratory abilities using the Boyden chamber (Figure 2A) showed that cells exposed to NaF at concentrations of 0.1–10 µM, both short- and long-term, exhibited increased migratory potential compared to the control cells. A higher concentration of NaF correlated with a greater tendency toward a migratory phenotype (2- to 5-fold increase) (Figure 2B). Statistical analysis within groups and between short- and long-term exposures did not reveal statistically significant differences (lowest *p* = 0.61). The “wound healing” assay indicated accelerated closure of the plate field under the influence of a 1 µM NaF solution compared to the control. The percentage of wound healing area was calculated, with the following results: 9.7% ± 5.7% for the control, 48.9% ± 9.1% for short exposure, and 88.3% ± 16.4% for long exposure to 1 µM NaF. Furthermore, long-term exposure to NaF led to faster wound closure compared to short-term exposure (Figure 2C).

### 2.3. Results of Flow Cytometry, Enzyme-Linked Immunosorbent Assays, and Zymography

**Figure 3:**
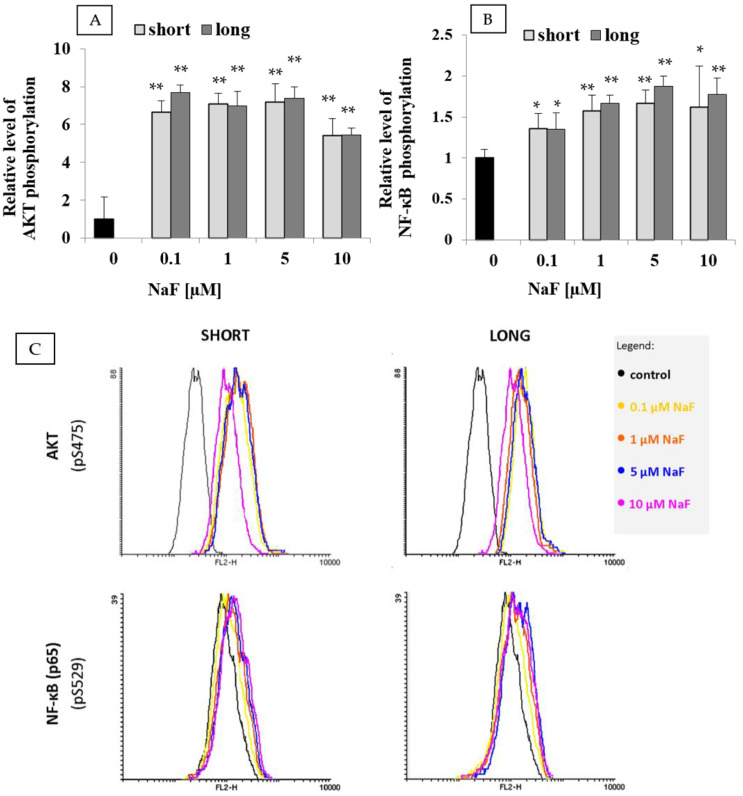
Levels of the phosphorylated forms of (**A**) AKT and (**B**) NF-κB (p65) in control and experimental conditions (0.1–10 µM NaF, t = 48 h). Data represent the arithmetic mean with standard deviations. Differences between passages were analyzed using the Mann–Whitney U test, and differences within groups were assessed with the Wilcoxon test (* *p* ≤ 0.05, ** *p* ≤ 0.01). (**C**) Representative FACS dot plot data for AKT and NF-κB (p65). Three biological and three technical replicates were performed.

Cytometric analysis of the phosphorylated (active) forms of AKT (Figure 3A) and NF-κB (Figure 3B) showed a significant increase in the activity of both factors. For AKT kinase, a substantial 6- to 7-fold increase in the level of phosphorylated AKT protein (pS473) was observed at all tested NaF concentrations compared to the control, with statistically significant differences. No statistically significant differences were found between exposure times at any NaF concentration.

For the NF-κB factor (Figure 3B), the phosphorylated form NF-κB p65 (pS529) increased approximately 1.4-, 1.7-, 1.9-, and 1.9-fold, respectively, at NaF concentrations of 0.1–10 µM compared to the control, with these increases being statistically significant. Differences between exposure durations were not statistically significant.

**Figure 4:**
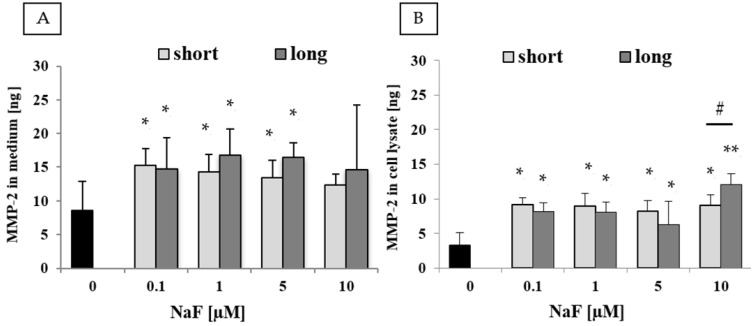
Quantity of MMP-2 in (**A**) culture medium and (**B**) cell lysate from control and experimental conditions (0.1–10 µM NaF, t = 48 h). Data represent the arithmetic mean with standard deviations. Differences between passages were analyzed using the Mann–Whitney U test (# *p* ≤ 0.05), and differences within groups were assessed using the Wilcoxon test (* *p* ≤ 0.05, ** *p* ≤ 0.01). Three biological and three technical replicates were performed.

Analysis of the medium after culturing U-87 cells exposed to NaF (Figure 4A) showed a significant increase in MMP-2 levels across all tested concentrations. Both short- and long-term exposure resulted in a statistically significant 1.5- to 2-fold increase in MMP-2 concentration in the medium compared to the control, except at 10 µM NaF, where the results were not statistically significant. No statistically significant differences were observed between short- and long-term exposure at any of the tested NaF concentrations.

Analysis of cell lysates from experimental setups exposed to NaF (Figure 4B) revealed a significant increase in MMP-2 levels at all tested concentrations. At 0.1–5 µM, both short- and long-term exposure led to approximately a 2.5- to 3-fold increase in MMP-2 levels compared to the control. Similar results were observed for cells exposed to 10 µM NaF under short-term exposure, whereas prolonged exposure (72 days) to 10 µM NaF resulted in a 4-fold increase in MMP-2 levels. All differences compared to the control were statistically significant for both short- and long-term exposure. At 10 µM NaF, the difference between short- and long-term exposure was also statistically significant.

**Figure 5:**
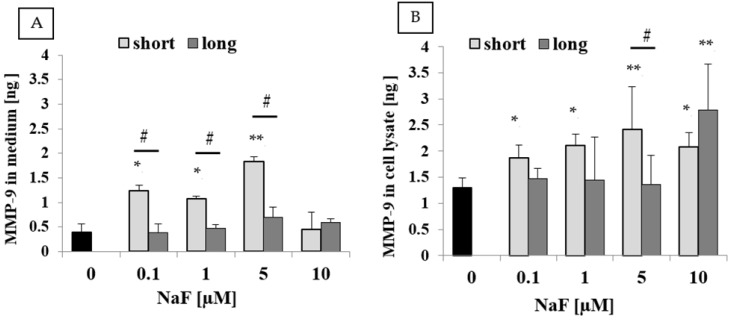
Quantity of MMP-9 in (**A**) culture medium and (**B**) cell lysate from control and experimental conditions (0.1–10 µM NaF, t = 48 h). Data represent the arithmetic mean with standard deviations. Differences between passages were analyzed using the Mann–Whitney U test (# *p* ≤ 0.05), and differences within groups were assessed using the Wilcoxon test (* *p* ≤ 0.05, ** *p* ≤ 0.01). Three biological and three technical replicates were performed.

Analysis of the medium from U-87 cells exposed to NaF (Figure 5A) revealed a significant increase in MMP-9 levels at concentrations of 0.1–5 µM for short-term exposure. Specifically, concentrations of 0.1 and 1 µM resulted in approximately a 3-fold increase in enzyme levels, while 5 µM led to a 4.5-fold increase. These differences compared to the control were statistically significant. For long-term exposure, MMP-9 levels were similar to those observed under control conditions. Comparative analysis between passages showed statistically significant differences between short- and long-term exposure at concentrations of 0.1–5 µM.

In cell lysates from U-87 cells exposed to NaF (Figure 5B), a significant increase in MMP-9 levels was observed across all NaF concentrations for short-term exposure (1.5- to 2-fold) and for long-term exposure only at 10 µM (approximately 2.5-fold). These differences were statistically significant compared to the control. For long-term exposure at NaF concentrations of 0.1–5 µM, no notable changes were observed compared to the control. Only at 5 µM NaF were the differences between short- and long-term exposure statistically significant.

**Figure 6:**
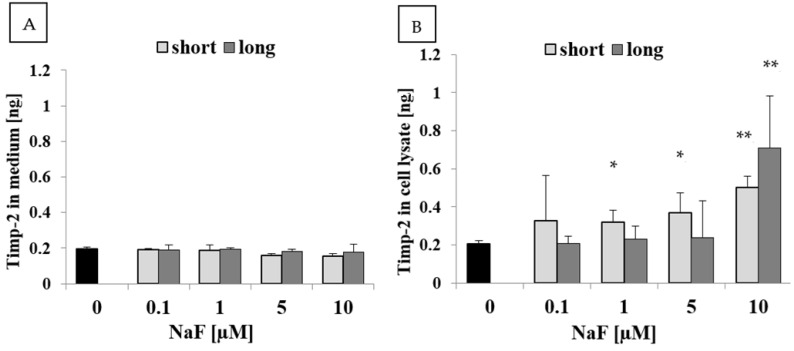
Quantity of Timp-2 in (**A**) culture medium and (**B**) cell lysate from control and experimental conditions (0.1–10 µM NaF, t = 48 h). Data represent the arithmetic mean with standard deviations. Differences between passages were analyzed using the Mann–Whitney U test, and differences within groups were assessed using the Wilcoxon test (* *p* ≤ 0.05, ** *p* ≤ 0.01). Three biological and three technical replicates were performed.

Analysis of the culture medium from U-87 cells exposed to NaF (Figure 6A) showed a slight decrease in Timp-2 levels within the 5–10 µM concentration range for short exposure. Under long exposure, Timp-2 levels remained similar to those in control conditions. Additionally, the comparison between passages did not reveal statistically significant differences between short and long exposure to NaF.

Analysis of U-87 cell lysates exposed to NaF (Figure 6B) indicated a clear increase in Timp-2 levels across all tested concentrations for short exposure (approximately 1.5- to 2.5-fold) and a significant increase at 10 µM for long exposure (approximately 3.5-fold). These differences were statistically significant, except at the 0.1 µM concentration. Within the 0.1–5 µM range, long exposure did not show notable changes compared to control conditions. Differences observed between short and long exposures were not statistically significant.

**Figure 7:**
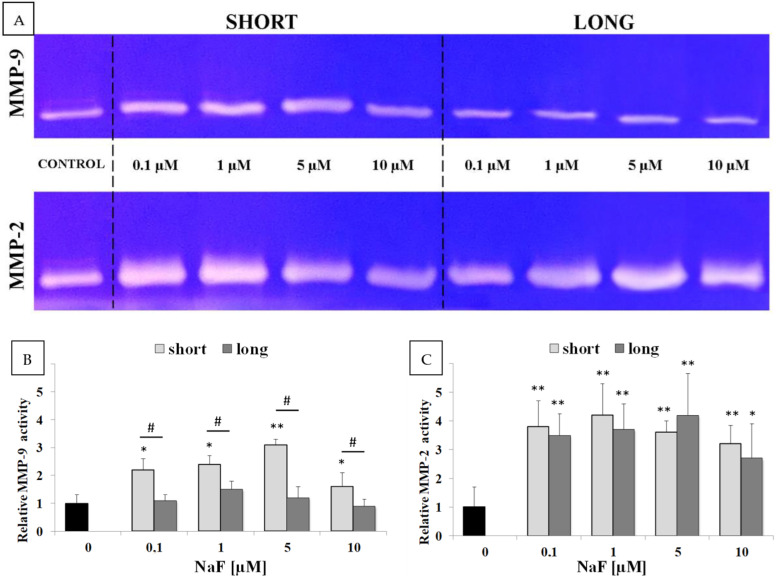
Representative zymogram images showing the activation of MMP-9 and MMP-2 metalloproteinases (**A**). Relative activity of MMP-9 (**B**) and MMP-2 (**C**) in culture medium from control and experimental setups (0.1–10 µM NaF, t = 48 h). Data represent the arithmetic mean with standard deviations. Differences between passages were analyzed using the Mann–Whitney U test (# *p* ≤ 0.05), and differences within groups were assessed using the Wilcoxon test (* *p* ≤ 0.05, ** *p* ≤ 0.01). Three biological and three technical replicates were performed.

Zymography revealed the presence of active MMP-9 (84 kDa), with negligible amounts of latent MMP-9 (92 kDa), as well as active MMP-2 (66 kDa) and small amounts of latent MMP-2 (72 kDa) in the post-culture medium of control cells (Figure 7A). Short-term exposure to NaF resulted in a marked increase in the active form of MMP-9, with the latent form detected only at 0.1 and 1 µM concentrations. A distinct increase in the active form of MMP-2 was also observed, with no detectable latent form across all tested conditions. Long-term exposure to NaF did not significantly alter the amount of active MMP-9, though no latent form band was observed. For MMP-2, a notable increase in the active form was observed, similar to short-term exposure, with no detectable latent form at any NaF concentration tested.

Densitometric analysis showed a statistically significant increase in MMP-2 activity for both short- and long-term exposure at all NaF concentrations. No statistically significant differences were observed between exposure durations (Figure 7C). This was not the case for MMP-9. Short-term exposure to NaF led to an increase in metalloproteinase activity (with statistically significant differences), whereas long-term exposure showed no effect (Figure 7B).

## 3. Discussion

Fluoride compounds are common environmental pollutants that, upon entering the human body, disrupt numerous physiological processes. The impact of fluoride on organs such as bones, liver, pancreas, lungs, heart, skeletal muscles, and kidneys is well-established [27]. Furthermore, the ability of fluorides to cross the blood–brain barrier (BBB) suggests that they may also disrupt metabolic processes in the central nervous system, as indicated by limited studies on fluoride’s effects on the brain [10]. However, there is a lack of well-documented studies demonstrating a direct impact of fluoride on brain tumor development, invasiveness, or resistance, including gliomas. Preliminary findings from in vitro studies on neuronal cell lines, in vivo studies in rodents, and observations from other tissues and organs, including human studies, tentatively raise questions and hypotheses regarding fluoride’s adverse effects in the context of brain tumor initiation and progression. These findings further suggest that both the direct effects of fluoride and its indirect effects on healthy cells and the tumor microenvironment may play a role in this process [26]. The negative impact of fluorides on the developing central nervous system, along with the increased susceptibility to brain tumors observed in children since the mid-1980s, should serve as an alarming signal.

### 3.1. Cytotoxicity of Fluorides on U-87 Cells

Cytotoxicity measurements within the range of concentrations used in this study indicated that NaF at concentrations of 0.1–10 µM does not inhibit U-87 cell growth. These results align with findings by Acra et al. (2012), where NaF showed no stimulatory or inhibitory effects on cell growth at low concentrations (0.125–1 mM) but reduced viable cell counts at higher concentrations, with complete cytotoxicity observed at 32 mM NaF. Notably, NaF exhibited greater cytotoxicity against glioma cell lines (T98G, U-87; IC_50_ = 1733–3350 µM) compared to normal oral epithelial cells (HGF, HPC, HPLF; CC_50_ = 4367–8350 µM) [28]. A similar trend was observed in recent studies on U-87 cells, indicating that glioma cells exhibit higher sensitivity to fluoride compounds compared to normal neuronal IMR-32 cells [29].

The present findings, together with existing literature, suggest that relatively low yet physiologically relevant concentrations of fluoride (up to 10 µM) do not induce cytotoxic effects; however, they may potentially impact the metabolism of GBM cells, including the U-87 line.

### 3.2. Migration Assays

Both the Boyden chamber assay and the “wound healing” assay revealed an increased migratory ability of U-87 cells across membranes and along plate surfaces. Cell motility increased with higher NaF concentrations in the medium, with incubation time further enhancing this effect. A similar study on pituitary tumor GH4C1 cells demonstrated comparable results, showing that NaF concentrations from 0.2 to 100 µM increased cell migration through the Boyden chamber in a concentration-dependent manner (higher concentrations correlated with greater cell migration) [30]. Unfortunately, no other scientific reports have presented similar observations. Nevertheless, our results demonstrate that biologically significant concentrations of fluoride can enhance the migration of cancer cells, suggesting that fluoride exposure may stimulate tumor invasion.

We hypothesize that the migratory capacity, rather than the proliferative potential, of NaF-treated U-87 cells was altered. In the MTT assay, at the NaF concentrations used, we did not observe an increase in formazan crystal formation compared to controls. This suggests that the cell count remains similar across all test conditions.

### 3.3. The Role of Fluorides in AKT Regulation

The results indicate that NaF at the applied concentrations strongly activates the AKT kinase pathway in U-87 cell lines, with a several-fold increase observed in both short- and long-term exposure conditions. AKT, as a central node in multiple signaling pathways, is recognized as a key regulator of growth and proliferation in malignant GBM cells [31]. It has been demonstrated that activation of the c-Met/AKT/mTOR signaling pathway in GBM upregulates mesenchymal markers, including SNAI1/2, vimentin, and Twist, while concurrently downregulating the epithelial marker E-cadherin [32]. Furthermore, AKT activation may enhance nuclear expression and transcriptional activity of SNAI1/2 through GSK-3β phosphorylation, thereby promoting GBM cell migration. Previous studies have shown that the AKT/GSK-3β/SNAI signaling pathway can induce a pro-invasive epithelial-to-mesenchymal transition (EMT) [33].

To date, no studies have confirmed or refuted the role of fluorides in regulating AKT activity in GBM cells; this study provides the first demonstration of this effect. Notably, most research indicates that fluoride compounds inhibit AKT activation, specifically acting upstream in the PI3K pathway [34,35,36,37,38,39]. However, these studies did not involve cancer cells and utilized much higher fluoride concentrations. The only study suggesting a pro-AKT role of fluoride involves fluoride nanoparticles of rare earth metals [40]. In this study, lanthanum fluoride (LaF_3_) and praseodymium fluoride (PrF_3_) in suspension stimulated the growth of cancer cells in three human tumor lines (lung—A549, colon—SW837, and breast—MCF7) through strong activation of the AKT and ERK pathways. Detailed analysis suggested that this may be due to mechanical stimulation of cell mechanosensors by fluoride nanoparticles [40]. This finding may be partly attributed to the high reactivity and electronegativity of fluoride anions.

### 3.4. Involvement of Fluorides in the Regulation of the NF-κB Pathway

Based on the presented results, it can be concluded that fluorides may influence the activation of the NF-κB transcription factor. The phosphorylated form of NF-κB p65 (pS529) increased by approximately 1.4-, 1.7-, 2-, and 1.9-fold at fluoride concentrations of 0.1, 1, 5, and 10 µM, respectively, compared to control, with these differences reaching statistical significance. No statistically significant differences were observed between short- and long-term NaF exposure, suggesting that NF-κB activity remains consistently elevated regardless of exposure duration. This sustained activity aligns with the previously observed constant activation of AKT kinase, a known component of the canonical NF-κB activation pathway in gliomas [41,42].

Currently, there are no studies confirming the impact of fluoride compounds on U-87 cells or other glioma cell lines. However, multiple in vitro and in vivo studies suggest an indirect role of fluorides in regulating the NF-κB pathway. Increased NF-κB expression induced by NaF has been observed in monocytes [43], macrophages [44], peripheral blood mononuclear cells [45], and human lung epithelial cell line (1.0–3.75 mM) [46]. Research in mice exposed to fluoride compounds revealed that NaF at doses exceeding 12 mg/kg body weight induced inflammatory reactions in the kidneys by activating NF-κB, reducing anti-inflammatory cytokines (IL-4 and IL-10), and increasing levels of PGE_2_, iNOS, COX-2, IL-6, and IL-8 compared to controls [47]. Another study in mice showed similar inflammation in the liver, linked to activation of the MAPK and NF-κB pathways and elevated levels of IL-1β, IL-6, IL-8, COX-2, and MCP-1 [48]. Similar findings were observed in the spleen [49]. Additionally, studies indicate that fluoride can activate the NF-κB pathway by promoting TNF-α synthesis [50] and inhibiting vitamin D receptor (VDR) expression, which typically reduces NF-κB activation [51,52]. Several studies also describe the effects of NaF on various brain tissues [53,54,55], though specific mechanisms remain unclear.

Our results appear to support existing literature, suggesting that fluoride compounds can activate NF-κB signaling pathways. Additional support may be inferred from other findings in this study, where increased MMP-2 and MMP-9 levels under NF-κB regulation suggest that low NaF concentrations in U-87 glioma cells might promote adverse NF-κB activation. Abnormal NF-κB activity is known to facilitate tumor invasion and impact therapeutic responses [56]. Neovascularization in GBM, essential for tumor growth, depends on several NF-κB target genes, including VEGF, IL-6, and IL-8 [57,58]. Furthermore, NF-κB regulates cancer cell invasion by controlling the expression of adhesion molecules such as fibronectin and vitronectin, which influence MMP-2 and MMP-9 activity [59,60]. NF-κB activation also promotes epithelial-to-mesenchymal transition (EMT), a crucial transformation for invasion and resistance [61]. Therefore, fluoride ions may act as an unfavorable factor, potentially stimulating pathways that enhance the progression and invasiveness of GBM.

### 3.5. The Role of Fluorides in Regulating the Activity of MMP-2/-9 and Timp-2

In this study, the effects of NaF on the levels of MMP-2 and MMP-9, as well as the tissue inhibitor of metalloproteinases, TIMP-2, in both cells and culture medium, were analyzed. A significant increase in the activity and quantity of both MMP-2 and MMP-9 was observed in NaF-treated cells after short-term exposure, while only MMP-2 showed a sustained increase following long-term exposure. Regarding the inhibitor TIMP-2, low NaF concentrations led to a slight elevation in its levels (more pronounced with short-term exposure) in both U-87 cells and culture medium. Notably, the highest NaF concentration (10 µM) induced an almost fourfold increase in TIMP-2 levels. It is well established that an imbalance favoring MMPs over TIMPs promotes matrix proteolysis, a process involved in various pathological conditions, including tumor invasion [62,63]. Elevated MMP-2 and MMP-9 activity is closely linked to the formation of metastatic niches, angiogenesis, and other functions within the tumor microenvironment, such as the chemotaxis of inflammatory cells and the perpetuation of an inflammatory milieu [24]. Increased TIMP-2 levels positively correlate with MMP-2 activation and poor prognoses in cancer patients, with a clear association between TIMP-2 levels and cancer cell migration [64]. These findings align with the observed results in this study, where low concentrations of NaF led to an increase in both the concentration (ELISA) and activity (zymography) of MMP-2 and MMP-9 in U-87 cells. These are the first results to suggest a potential role for fluoride compounds in promoting the invasive potential of GBM cells and cancer cells more broadly.

However, several reports suggest that fluorides, depending on their concentration, may alter the concentration and activity of MMPs in various tissues. For instance, low doses have been shown to slightly increase MMP-2 and MMP-9 activity in mouse preosteoblast MC3T3-E1 cells after 24 h [65]. Administering NaF to rats in situ (150 mg/L) led to a significant increase in MMP-9 expression and protein levels in uterine tissues [66]. Additionally, elevated MMP-9 expression, protein levels, and IL-17 concentrations were observed in cardiac muscle [67]. Chronic exposure to fluoride has also been shown to increase MMP-9 expression, cause blood–brain barrier (BBB) disruption, and induce changes in neurocytes [68]. Moreover, it disrupts the balance in the expression and levels of MMP-2, MMP-9, and their inhibitors (TIMP-2 and TIMP-3) in brain structures, including the cerebellum, striatum, prefrontal cortex, and hippocampus [53].

## 4. Materials and Methods

### 4.1. Cell Cultures

Experimental work was conducted using the human glioblastoma U-87 cell line (Sigma-Aldrich, St. Louis, MO, USA). Cells were cultured in EMEM medium (EBSS, Sigma-Aldrich) supplemented with 10% FBS (Sigma-Aldrich), 2 mM glutamine (Sigma-Aldrich), 1% NEAA (Sigma-Aldrich), 1 mM sodium pyruvate (Sigma-Aldrich), and antibiotics (penicillin [100 IU/mL] and streptomycin [10 μg/mL], Sigma-Aldrich) under optimal conditions (37 °C, 5% CO_2_ atmosphere, 95% humidity).

High-passage cell lines often exhibit altered responses to stimuli, growth rates, protein expression, and transfection efficiency compared to low-passage cells. To minimize passage-related effects in our experiments, we limited the number of passages during prolonged NaF exposure [69].

The U-87 cell line was cultured under two fluoride exposure regimes: short-term (1 passage, 3 days of exposure) and long-term (24 passages, 72 days of exposure). The final concentrations of sodium fluoride (NaF, Sigma-Aldrich) in the culture wells were 0.1 µM, 1 µM, 5 µM, and 10 µM, chosen based on fluoride concentrations measured in human serum from regions with varying levels of fluoride exposure [70,71,72,73]. U-87 cells pre-exposed to NaF (short- or long-term) in culture flasks were collected for further experiments and seeded in appropriate culture plates, with NaF concentrations maintained at 0.1–10 µM. Depending on the analysis, incubation time was extended by an additional 12 or 48 h in the presence of NaF. Cells were then detached using either 0.25% trypsin (Sigma-Aldrich) or a cell scraper (BIOLOGIX, Camarillo, CA, USA), depending on the type of analysis, as trypsinization may affect cell surface proteins.

### 4.2. Cell Viability Test (MTT Method)

Analyses were performed prior to exposing cells to NaF (short- and long-term). The cytostatic/cytotoxic effects of NaF were assessed by determining the percentage of growth inhibition in U-87 cells treated with NaF at concentrations ranging from 0.1 to 2500 µM, relative to untreated control cells. NaF incubation was conducted for 72 h, after which 20 µL of fresh MTT solution (5 mg/mL) was added to each well. Following a 2 h incubation with MTT under optimal conditions, the medium was removed, and formazan crystals were dissolved with 200 µL of DMSO (Sigma-Aldrich) per well. Spectrophotometric measurements were taken using a UV-M340 microplate reader (Asys Hitachi, Tokyo, Japan) with MicroWin 2000 software, and results were exported to Microsoft Excel.

### 4.3. Cell Mobility—"Wound Healing Assay”

After pre-exposure to NaF (short- or long-term), U-87 MG cell suspensions were seeded onto 6-well plates (controls and 1 µM NaF with short and long exposure, 20,000 cells/cm^2^). After 72 h (control or with 1 µM NaF, 70–80% confluence), cell layers were scratched with 200 µL pipette tips and washed with PBS to remove detached and damaged cells. Fresh serum-free EMEM medium (control or with 1 µM NaF) was added to each well, and wound closure was assessed after 12 h using a light microscope. The percentage of wound closure was calculated using ZEN 3.7 Microscopy Software.

### 4.4. Cell Migration Potential (Boyden Chamber)

To assess the migratory potential of U-87 cells in control and experimental conditions (0.1–10 µM NaF), a modified Boyden Chamber migration assay was used, with 10% FBS as the chemoattractant. U-87 cells, suspended in EMEM medium without FBS (control and 0.1–10 µM NaF, no vehicle), were seeded at a density of 10^5^ cells in the upper chamber of a Transwell system with 8.0 μm pores (Thermo Fisher Scientific). After 12 h of incubation, non-migrating cells on the upper membrane surface were removed, and cells on the lower surface were fixed with 4% paraformaldehyde and stained with Giemsa (Merck, Darmstadt, Germany). Stained cells were photographed and counted under a microscope using ZEN 3.7 Microscopy Software.

### 4.5. AKT Kinase (pS473)

After pre-exposure to NaF (short- or long-term), cells were seeded in 24-well plates and incubated for 48 h under control conditions or with specified NaF concentrations. Cells were then detached using 0.25% trypsin, fixed with BD Cytofix Fixation Buffer (Becton Dickinson, Franklin Lakes, NJ, USA) for 10 min, and permeabilized on ice for 30 min with BD Phosflow Perm Buffer III (Becton Dickinson, Franklin Lakes, NJ, USA). Cells were subsequently resuspended in a staining solution containing an M89-61 antibody specific for the phosphorylated form of AKT at S473, conjugated to R-phycoerythrin (Becton Dickinson). Following a 50 min incubation (in the dark at 2–8 °C), cells were washed twice with Stain Buffer (Becton Dickinson) and analyzed by flow cytometry (FACScan; Becton Dickinson, Franklin Lakes, NJ, USA). Green fluorescence (FL-1) was recorded with a 496 ± 15 nm laser (λ_ex = 576 nm) for 10^4^ cells per sample.

### 4.6. NF-κB p65 (pS529)

After pre-exposure to NaF (short- or long-term), cells were seeded in 24-well plates and incubated for 48 h under control conditions or with specified NaF concentrations. Cells were then detached using 0.25% trypsin, collected, and fixed with BD Cytofix Fixation Buffer (Becton Dickinson) for 10 min at 37 °C, followed by permeabilization on ice for 30 min using BD Phosflow Perm Buffer III (Becton Dickinson). Subsequently, cells were suspended in a staining solution containing the K10-895.12.50 monoclonal antibody, which recognizes phosphorylated serine 529 (pS529) in the transactivation domain of the human NF-κB p65 subunit, conjugated with phycoerythrin (PE) (Becton Dickinson). Following a 90 min incubation (in the dark, at 2–8 °C), cells were washed twice with Stain Buffer (Becton Dickinson) and analyzed by flow cytometry (FACScan; Becton Dickinson). Green fluorescence (FL-1) was recorded using a 496 ± 15 nm laser (λ_ex = 576 nm) for 10^4^ cells per sample.

### 4.7. Enzyme-Linked Immunosorbent Assays (ELISA)

To quantify protein concentrations, commercial ELISA assays were used, following the manufacturer’s instructions. Cell lysates (prepared by sonication and freeze-thaw cycles) and culture media from individual setups were analyzed. Results from each sample were normalized and calculated per 1 million cells for each experimental condition. Measurements were conducted with a UVA 340 spectrophotometer (ASYS) in at least three biological and technical replicates. The following proteins were assessed using this method: total matrix metalloproteinase 2 (MMP-2) using the Human MMP-2 ELISA Kit (EH0017, Fine-Test); total matrix metalloproteinase 9 (MMP-9) using the Human MMP-9 ELISA Kit (EH0238, Fine-Test); tissue inhibitor of metalloproteinases 2 (TIMP-2) using the Human TIMP-2 ELISA Kit (EH0295, Fine-Test).

### 4.8. Zymography

To prepare samples for zymography, U-87 cells were cultured in medium without FBS for 24 h prior to the experiment. Post-culture medium samples were mixed with loading buffer in a 3:1 ratio and incubated for 30 min at room temperature. Then, 20 µL of each sample (standard, control, and NaF-treated) was loaded onto polyacrylamide gels containing gelatin. The gels were placed in a vertical electrophoresis system, filled with electrode buffer to the appropriate level, and run at 4 °C under controlled separation speed.

After electrophoresis, the gels were immersed in renaturing solution (2.5% Triton X-100, Sigma-Aldrich) for 30 min at room temperature on a shaker. Subsequently, the gels were transferred to incubation buffer and placed in an incubator at 37 °C on a rocker for 12 h. Following incubation, the gels were stained with 0.5% Coomassie Brilliant Blue (Sigma-Aldrich) for 60 min at room temperature on a rocker. The stained gels were then washed three times with destaining solution (methanol/acetic acid/H_2_O in a 3:1:6 ratio) and analyzed densitometrically using an iBright 1500 transilluminator (Thermo Fisher Scientific).

### 4.9. Statistical Analysis

Statistical analysis of the obtained results was conducted using Statistica 13.3 software. After examining the distribution of data, which deviated from the normal distribution, non-parametric tests were used for further analysis. To investigate differences between short and long exposure at a given NaF concentration, the Mann–Whitney U test was applied (#—*p* ≤ 0.05; ##—*p* ≤ 0.01). To examine differences within a specific group, between individual concentrations and the control, the Wilcoxon test was used (*—*p* ≤ 0.05; **—*p* ≤ 0.01; ***—*p* ≤ 0.001).

## 5. Conclusions

The results of this study present compelling data suggesting that biologically relevant concentrations of fluoride compounds may enhance the migration of U-87 glioblastoma cells, indicating a potential role of fluoride exposure in stimulating the invasive potential of these cells. The observed increases in AKT kinase activation, transcription factors such as NF-κB, secretion of matrix metalloproteinases MMP-2 and MMP-9, and their enhanced activation, together with migration assays, support the hypothesis that fluoride compounds may promote a pro-invasive phenotype in glioblastoma multiforme cells.

The limited scope of research in this area precludes definitive conclusions; however, in light of the present findings and available literature, it is plausible that fluorides could modulate cancer cell signaling pathways associated with invasiveness. Physiologically high concentrations of fluorides may not only exert toxicity on healthy tissues but also influence transformed cancer cells, interacting both directly with these cells and with their microenvironment. This interaction may create a niche in which, for instance, GBM cells could more effectively infiltrate adjacent tissues and potentially metastasize beyond the primary tumor site.

The findings of this study contribute to existing knowledge and may guide further research on cellular and animal models. Additionally, future studies could enhance public awareness of the toxic effects of fluoride compounds on human health, particularly regarding the central nervous system, and inform preventive strategies for individuals at risk of fluoride exposure.

## Data Availability

The datasets generated during and analyzed during the current study are not publicly available due to they are part of PhD thesis but are available from the corresponding author upon reasonable request.

## Abbreviations

AKT—protein kinase B, BBB—blood–brain barrier, CNS—central nervous system, COX-2—cyclooxygenase 2, ECM—extracellular matrix, EMT—epithelial to mesenchymal transition, FBS—bovine serum, GBM—glioblastoma multiforme, IL—interleukin, iNOS—inducible nitric oxide synthase, MCP-1—monocyte chemoattractant protein 1, MMPs—matrix metalloproteinases, NaF—sodium fluoride, NF-κB—nuclear factor-kappa B, PE—phycoerythrin, PGE2—prostaglandin E2, TIMP—tissue inhibitor of metalloproteinases, TNF-α—tumor necrosis factor α, VDR—vitamin D receptor, VEGF—vascular endothelial growth factor.

## References

[1] Strunecka A., Strunecky O. (2020). Mechanisms of Fluoride Toxicity: From Enzymes to Underlying Integrative Networks. Appl. Sci..

[2] Jha S.K., Mishra V.K., Sharma D.K., Damodaran T., Whitacre D.M. (2011). Fluoride in the Environment and Its Metabolism in Humans. Reviews of Environmental Contamination and Toxicology.

[3] Medjedovic E., Medjedovic S., Deljo D., Sukalo A. (2015). Impact of Fluoride on Dental Health Quality. Mater. Socio Medica.

[4] World Health Organization (2019). Preventing Disease Through Healthy Environments: Inadequate or Excess Fluoride: A Major Public Health Concern.

[5] O’Mullane D.M., Baez R.J., Jones S., Lennon M.A., Petersen P.E., Rugg-Gunn A.J., Whelton H., Whitford G.M. (2016). Fluoride and Oral Health. Community Dent. Health.

[6] Bombik E., Bombik A., Rymuza K. (2020). The Influence of Environmental Pollution with Fluorine Compounds on the Level of Fluoride in Soil, Feed and Eggs of Laying Hens in Central Pomerania, Poland. Environ. Monit. Assess..

[7] Ghanbarian M., Ghanbarian M., Tabatabaie T., Ghanbarian M., Ghadiri S.-K. (2021). Distributing and Assessing Fluoride Health Risk in Urban Drinking Water Resources in Fars Province, Iran, Using the Geographical Information System. Environ. Geochem. Health.

[8] Jaudenes J.R., Gutiérrez Á.J., Paz S., Rubio C., Hardisson A. (2020). Fluoride Risk Assessment from Consumption of Different Foods Commercialized in a European Region. Appl. Sci..

[9] Riddell J.K., Malin A.J., McCague H., Flora D.B., Till C. (2021). Urinary Fluoride Levels among Canadians with and without Community Water Fluoridation. Int. J. Environ. Res. Public Health.

[10] Grandjean P. (2019). Developmental Fluoride Neurotoxicity: An Updated Review. Environ. Health.

[11] Liu Y.-J., Gao Q., Wu C.-X., Guan Z.-Z. (2010). Alterations of nAChRs and ERK1/2 in the Brains of Rats with Chronic Fluorosis and Their Connections with the Decreased Capacity of Learning and Memory. Toxicol. Lett..

[12] Reddy P., Reddy K., Kumar K. (2011). Neurodegenerative Changes in Different Regions of Brain, Spinal Cord and Sciatic Nerve of Rats Treated with Sodium Fluoride. J. Med. Allied Sci..

[13] Wu C., Gu X., Ge Y., Jianhai Z., Wang J. (2006). Effects of High Fluoride and Arsenic on Brain Biochemical Indexes and Learning-Memory in Rats. Fluoride.

[14] Bartos M., Gumilar F., Gallegos C.E., Bras C., Dominguez S., Cancela L.M., Minetti A. (2019). Effects of Perinatal Fluoride Exposure on Short- and Long-Term Memory, Brain Antioxidant Status, and Glutamate Metabolism of Young Rat Pups. Int. J. Toxicol..

[15] Kupnicka P., Listos J., Tarnowski M., Kolasa-Wołosiuk A., Wąsik A., Łukomska A., Barczak K., Gutowska I., Chlubek D., Baranowska-Bosiacka I. (2020). Fluoride Affects Dopamine Metabolism and Causes Changes in the Expression of Dopamine Receptors (D1R and D2R) in Chosen Brain Structures of Morphine-Dependent Rats. Int. J. Mol. Sci..

[16] Sun Z., Zhang Y., Xue X., Niu R., Wang J. (2018). Maternal Fluoride Exposure during Gestation and Lactation Decreased Learning and Memory Ability, and Glutamate Receptor mRNA Expressions of Mouse Pups. Hum. Exp. Toxicol..

[17] Lopes G.O., Martins Ferreira M.K., Davis L., Bittencourt L.O., Bragança Aragão W.A., Dionizio A., Rabelo Buzalaf M.A., Crespo-Lopez M.E., Maia C.S.F., Lima R.R. (2020). Effects of Fluoride Long-Term Exposure over the Cerebellum: Global Proteomic Profile, Oxidative Biochemistry, Cell Density, and Motor Behavior Evaluation. Int. J. Mol. Sci..

[18] Guan Z.-Z., Wang Y.-N., Xiao K.-Q., Dai D.-Y., Chen Y.-H., Liu J.-L., Sindelar P., Dallner G. (1998). Influence of Chronic Fluorosis on Membrane Lipids in Rat Brain. Neurotoxicol. Teratol..

[19] Claus E.B., Walsh K.M., Wiencke J., Molinaro A.M., Wiemels J.L., Schildkraut J.M., Bondy M.L., Berger M., Jenkins R., Wrensch M. (2015). Survival and Low Grade Glioma: The Emergence of Genetic Information. Neurosurg. Focus.

[20] Kanderi T., Gupta V. (2021). Glioblastoma Multiforme. StatPearls.

[21] Arora A., Somasundaram K. (2019). Glioblastoma vs Temozolomide: Can the Red Queen Race Be Won?. Cancer Biol. Ther..

[22] Chopra S., Overall C.M., Dufour A. (2019). Matrix Metalloproteinases in the CNS: Interferons Get Nervous. Cell. Mol. Life Sci..

[23] Paw I., Carpenter R.C., Watabe K., Debinski W., Lo H.-W. (2015). Mechanisms Regulating Glioma Invasion. Cancer Lett..

[24] Roomi M.W., Kalinovsky T., Rath M., Niedzwiecki A. (2017). Modulation of MMP-2 and MMP-9 Secretion by Cytokines, Inducers and Inhibitors in Human Glioblastoma T-98G Cells. Oncol. Rep..

[25] Zhou W., Yu X., Sun S., Zhang X., Yang W., Zhang J., Zhang X., Jiang Z. (2019). Increased Expression of MMP-2 and MMP-9 Indicates Poor Prognosis in Glioma Recurrence. Biomed. Pharmacother..

[26] Żwierełło W., Maruszewska A., Skórka-Majewicz M., Gutowska I. (2023). Fluoride in the Central Nervous System and Its Potential Influence on the Development and Invasiveness of Brain Tumours—A Research Hypothesis. Int. J. Mol. Sci..

[27] Skórka-Majewicz M., Goschorska M., Żwierełło W., Baranowska-Bosiacka I., Styburski D., Kapczuk P., Gutowska I. (2020). Effect of Fluoride on Endocrine Tissues and Their Secretory Functions—Review. Chemosphere.

[28] Acra A.M., Sakagami H., Matsuta T., Adachi K., Otsuki S., Nakajima H., Koh T., Machino M., Ogihara T., Watanabe K. (2012). Effect of Three Fluoride Compounds on the Growth of Oral Normal and Tumor Cells. Vivo.

[29] Puty B., Bittencourt L.O., Nogueira I.C., Buzalaf M.A.R., Oliveira E.H., Lima R.R. (2021). Human cultured IMR-32 neuronal-like and U87 glial-like cells have different patterns of toxicity under fluoride exposure. PLoS ONE.

[30] Mendoza-Schulz A., Solano-Agama C., Arreola-Mendoza L., Reyes-Márquez B., Barbier O., Del Razo L.M., Mendoza-Garrido M.E. (2009). The Effects of Fluoride on Cell Migration, Cell Proliferation, and Cell Metabolism in GH4C1 Pituitary Tumour Cells. Toxicol. Lett..

[31] Xie C., Zhou M., Lin J., Wu Z., Ding S., Luo J., Zhan Z., Cai Y., Xue S., Song Y. (2020). EEF1D Promotes Glioma Proliferation, Migration, and Invasion through EMT and PI3K/Akt Pathway. BioMed Res. Int..

[32] Jia Y., Feng Q., Tang B., Luo X., Yang Q., Yang H., Li Q. (2021). Decorin Suppresses Invasion and EMT Phenotype of Glioma by Inducing Autophagy via C-Met/Akt/mTOR Axis. Front. Oncol..

[33] Wang N., Song Q., Yu H., Bao G. (2021). Overexpression of FBXO17 Promotes the Proliferation, Migration and Invasion of Glioma Cells Through the Akt/GSK-3β/Snail Pathway. Cell Transplant..

[34] Fujiwara N., Whitford G.M., Bartlett J.D., Suzuki M. (2021). Curcumin Suppresses Cell Growth and Attenuates Fluoride-Mediated Caspase-3 Activation in Ameloblast-like LS8 Cells. Environ. Pollut..

[35] Kuang P., Deng H., Liu H., Cui H., Fang J., Zuo Z., Deng J., Li Y., Wang X., Zhao L. (2018). Sodium Fluoride Induces Splenocyte Autophagy via the Mammalian Targets of Rapamycin (mTOR) Signaling Pathway in Growing Mice. Aging.

[36] Ma L., Zhang R., Li D., Qiao T., Guo X. (2021). Fluoride Regulates Chondrocyte Proliferation and Autophagy via PI3K/AKT/mTOR Signaling Pathway. Chem. Biol. Interact..

[37] Wei M., Duan D., Liu Y., Wang Z., Li Z. (2014). Autophagy May Protect MC3T3-E1 Cells from Fluoride-Induced Apoptosis. Mol. Med. Rep..

[38] Xu L., Deng C., Zhang Y., Zhao L., Linghu Y., Yu Y. (2021). Expression of Autophagy-Related Factors LC3A and Beclin 1 and Apoptosis-Related Factors Bcl-2 and BAX in Osteoblasts Treated with Sodium Fluoride. Front. Physiol..

[39] Zhou B.-H., Tan P.-P., Jia L.-S., Zhao W.-P., Wang J.-C., Wang H.-W. (2018). PI3K/AKT Signaling Pathway Involvement in Fluoride-Induced Apoptosis in C2C12 cells. Chemosphere.

[40] Semashko V.V., Pudovkin M.S., Cefalas A.-C., Zelenikhin P.V., Gavriil V.E., Nizamutdinov A.S., Kollia Z., Ferraro A., Sarantopoulou E. (2018). Tiny Rare-Earth Fluoride Nanoparticles Activate Tumour Cell Growth via Electrical Polar Interactions. Nanoscale Res. Lett..

[41] Jin S., Li X., Dai Y., Li C., Wang D. (2020). NF-κB-Mediated miR-650 Plays Oncogenic Roles and Activates AKT/ERK/NF-κB Pathways by Targeting RERG in Glioma Cells. Cell. Oncol..

[42] Xu H.-B., Chen X.-Z., Yu Z.-L., Xue F. (2023). Guggulsterone from Commiphora Mukul Potentiates Anti-Glioblastoma Efficacy of Temozolomide In Vitro and In Vivo via down-Regulating EGFR/PI3K/Akt Signaling and NF-κB Activation. J. Ethnopharmacol..

[43] Stachowska E., Gutowska I., Bober J., Grymula K., Dziedziejko V., Chlubek D. (2005). Sodium fluoride enhancement of monocyte differentiation via nuclear factor κb mechanism. Res. Rep..

[44] Tian Y., Huo M., Li G., Li Y., Wang J. (2016). Regulation of LPS-Induced mRNA Expression of pro-Inflammatory Cytokines via Alteration of NF-κB Activity in Mouse Peritoneal Macrophages Exposed to Fluoride. Chemosphere.

[45] Chen Q., Wang Z., Xiong Y., Zou X., Liu Z. (2010). Comparative Study of P38 MAPK Signal Transduction Pathway of Peripheral Blood Mononuclear Cells from Patients with Coal-Combustion-Type Fluorosis with and without High Hair Selenium Levels. Int. J. Hyg. Environ. Health.

[46] Refsnes M., Skuland T., Schwarze P., Lag M., Ovrevik J. (2014). Differential NF-κB and MAPK Activation Underlies Fluoride- and TPA-Mediated CXCL8 (IL-8) Induction in Lung Epithelial Cells. J. Inflamm. Res..

[47] Luo Q., Cui H., Deng H., Kuang P., Liu H., Lu Y., Fang J., Zuo Z., Deng J., Li Y. (2017). Sodium Fluoride Induces Renal Inflammatory Responses by Activating NF-κB Signaling Pathway and Reducing Anti-Inflammatory Cytokine Expression in Mice. Oncotarget.

[48] Chen L., Kuang P., Liu H., Wei Q., Cui H., Fang J., Zuo Z., Deng J., Li Y., Wang X. (2019). Sodium Fluoride (NaF) Induces Inflammatory Responses Via Activating MAPKs/NF-κB Signaling Pathway and Reducing Anti-Inflammatory Cytokine Expression in the Mouse Liver. Biol. Trace Elem. Res..

[49] Deng H., Kuang P., Cui H., Luo Q., Liu H., Lu Y., Fang J., Zuo Z., Deng J., Li Y. (2017). Sodium Fluoride Induces Apoptosis in Mouse Splenocytes by Activating ROS-Dependent NF-κB Signaling. Oncotarget.

[50] Sana S., Ghosh S., Das N., Sarkar S., Mandal A.K. (2017). Vesicular Melatonin Efficiently Downregulates Sodium Fluoride-Induced Rat Hepato- and Broncho-TNF-α, TGF-β Expressions, and Associated Oxidative Injury: A Comparative Study of Liposomal and Nanoencapsulated Forms. Int. J. Nanomed..

[51] Tiwari S., Gupta S.K., Kumar K., Trivedi R., Godbole M.M. (2004). Simultaneous Exposure of Excess Fluoride and Calcium Deficiency Alters VDR, CaR, and Calbindin D 9 k mRNA Levels in Rat Duodenal Mucosa. Calcif. Tissue Int..

[52] Sun J., Kong J., Duan Y., Szeto F.L., Liao A., Madara J.L., Li Y.C. (2006). Increased NF-kappaB Activity in Fibroblasts Lacking the Vitamin D Receptor. Am. J. Physiol. Endocrinol. Metab..

[53] Łukomska A., Baranowska-Bosiacka I., Dec K., Pilutin A., Tarnowski M., Jakubczyk K., Żwierełło W., Skórka-Majewicz M., Chlubek D., Gutowska I. (2021). Changes in Gene and Protein Expression of Metalloproteinase-2 and -9 and Their Inhibitors TIMP2 and TIMP3 in Different Parts of Fluoride-Exposed Rat Brain. Int. J. Mol. Sci..

[54] Zhang J., Zhu W.-J., Xu X.-H., Zhang Z.-G. (2011). Effect of Fluoride on Calcium Ion Concentration and Expression of Nuclear Transcription Factor Kappa-B Ρ65 in Rat Hippocampus. Exp. Toxicol. Pathol. Off. J. Ges. Toxikol. Pathol..

[55] Zhang M., Wang A., Xia T., He P. (2008). Effects of Fluoride on DNA Damage, S-Phase Cell-Cycle Arrest and the Expression of NF-kappaB in Primary Cultured Rat Hippocampal Neurons. Toxicol. Lett..

[56] Raychaudhuri B., Han Y., Lu T., Vogelbaum M.A. (2007). Aberrant Constitutive Activation of Nuclear Factor kappaB in Glioblastoma Multiforme Drives Invasive Phenotype. J. Neurooncol..

[57] Fianco G., Mongiardi M.P., Levi A., De Luca T., Desideri M., Trisciuoglio D., Del Bufalo D., Cinà I., Di Benedetto A., Mottolese M. (2017). Caspase-8 Contributes to Angiogenesis and Chemotherapy Resistance in Glioblastoma. eLife.

[58] Jiang L., Song L., Wu J., Yang Y., Zhu X., Hu B., Cheng S.-Y., Li M. (2013). Bmi-1 Promotes Glioma Angiogenesis by Activating NF-κB Signaling. PLoS ONE.

[59] Ritchie C.K., Giordano A., Khalili K. (2000). Integrin Involvement in Glioblastoma Multiforme: Possible Regulation by NF-kappaB. J. Cell. Physiol..

[60] Zhang J.-F., Wang P., Yan Y.-J., Li Y., Guan M.-W., Yu J.-J., Wang X.-D. (2017). IL-33 Enhances Glioma Cell Migration and Invasion by Upregulation of MMP2 and MMP9 via the ST2-NF-κB Pathway. Oncol. Rep..

[61] Liu T., Ma W., Xu H., Huang M., Zhang D., He Z., Zhang L., Brem S., O’Rourke D.M., Gong Y. (2018). PDGF-Mediated Mesenchymal Transformation Renders Endothelial Resistance to Anti-VEGF Treatment in Glioblastoma. Nat. Commun..

[62] Fischer T., Riedl R. (2019). Inhibitory Antibodies Designed for Matrix Metalloproteinase Modulation. Molecules.

[63] Raeeszadeh-Sarmazdeh M., Coban M., Sankaran B., Radisky E. (2020). Engineering Protein Therapeutics for Cancer Based on the Natural Matrix Metalloproteinase Inhibitor TIMP-1. FASEB J..

[64] Sounni N.E., Rozanov D.V., Remacle A.G., Golubkov V.S., Noel A., Strongin A.Y. (2010). Timp-2 Binding with Cellular MT1-MMP Stimulates Invasion-Promoting MEK/ERK Signaling in Cancer Cells. Int. J. Cancer.

[65] Slompo C., Buzalaf C.P., Damante C.A., Martins G.M., Hannas A.R., Buzalaf M.A.R., Oliveira R.C. (2012). Fluoride Modulates Preosteoblasts Viability and Matrix Metalloproteinases-2 and -9 Activities. Braz. Dent. J..

[66] Wang H., Zhao W., Tan P., Liu J., Zhao J., Zhou B. (2017). The MMP-9/TIMP-1 System Is Involved in Fluoride-Induced Reproductive Dysfunctions in Female Mice. Biol. Trace Elem. Res..

[67] Quadri J.A., Sarwar S., Kar P., Singh S., Mallick S.R., Arava S., Nag T.C., Roy T.S., Shariff A. (2018). Fluoride Induced Tissue Hypercalcemia, IL-17 Mediated Inflammation and Apoptosis Lead to Cardiomyopathy: Ultrastructural and Biochemical Findings. Toxicology.

[68] Qing-Feng S., Ying-Peng X., Tian-Tong X. (2019). Matrix Metalloproteinase-9 and P53 Involved in Chronic Fluorosis Induced Blood-Brain Barrier Damage and Neurocyte Changes. Arch. Med. Sci..

[69] ATCC Passage Number Effects in Cell Lines. https://www.atcc.org/resources/technical-documents/passage-number-effects-in-cell-lines.

[70] DenBesten P., Li W. (2011). Chronic Fluoride Toxicity: Dental Fluorosis. Monogr. Oral Sci..

[71] Gillespie G., Jackson Rudd D., Zhang S., Schaeffer A., Tomek C., Larson P., Stoch S.A., Iwamoto M. (2022). Fluoride Pharmacokinetics in Urine and Plasma Following Multiple Doses of MK-8507, an Investigational, Oral, Once-Weekly Nonnucleoside Reverse Transcriptase Inhibitor. J. Clin. Pharmacol..

[72] Gillespie G., Rudd D.J., Zhang S., Schaeffer A., Tomek C., Larson P., Stoch S.A., Iwamoto M. (2021). A Phase 1 Trial to Evaluate the Relationship Between Fluoride Intake and Urinary Fluoride Excretion in Healthy Participants. J. Clin. Pharmacol..

[73] Guth S., Hüser S., Roth A., Degen G., Diel P., Edlund K., Eisenbrand G., Engel K.-H., Epe B., Grune T. (2020). Toxicity of Fluoride: Critical Evaluation of Evidence for Human Developmental Neurotoxicity in Epidemiological Studies, Animal Experiments and in Vitro Analyses. Arch. Toxicol..

